# Choice of lipid supplementation for in vitro erythroid cell culture impacts reticulocyte yield and characteristics

**DOI:** 10.1038/s41598-026-37229-z

**Published:** 2026-01-29

**Authors:** C. M. Freire, N. R. King, M. Dzieciatkowska, D. Stephenson, J. G. G. Dobbe, G. J. Streekstra, A. D’Alessandro, T. J. Satchwell, A. M. Toye

**Affiliations:** 1 School of Biochemistry and Cellular Molecular Medicine, University of Bristol, Bristol, UK; 2 Department of Biochemistry and Molecular Genetics, University of Colorado Anschutz Medical Campus, Aurora, CO 80045 USA; 3 Biomedical Engineering and Physics, Amsterdam UMC location University of Amsterdam, Meibergdreef 9, Amsterdam, The Netherlands; 4 Centre for Biomedical Research, School of Applied Sciences, University of the West of England, Bristol, UK

**Keywords:** Biochemistry, Biological techniques, Biotechnology, Cell biology

## Abstract

Lipids, particularly cholesterol, are critical components of red blood cell (RBC) membranes, influencing protein function, cell stability, and deformability. Reticulocytes (young RBC) derived from *in vitro* erythroid cultures have been reported to possess less cholesterol than their native counterparts, compromising their functional integrity and lifespan. However, variability in starting materials and culture protocols between studies has hindered definitive conclusions regarding the nature and consequences of this lipid deficiency. Here, we evaluated the influence of lipid sources on reticulocyte quality using a well-established CD34⁺ erythroid culture system. We compared the use of human AB serum and Octaplas (solvent/detergent (S/D)-extracted pooled plasma) as lipid sources. Our results reveal that S/D-extracted plasma leads to cholesterol-deficient reticulocytes with impaired characteristics, including reduced filtration yield, heightened osmotic fragility, and altered PIEZO1 activity. In contrast, AB serum supported the generation of functionally stable reticulocytes, with cholesterol supplementation required to rescue the majority of defects observed with culturing erythroid cells with plasma alone. Importantly, this study provides the first integrated lipidomic, metabolomic, and proteomic characterisation of *in vitro*-derived reticulocytes cultured under distinct lipid conditions. These multi-omic datasets offer new insights into the consequences of reduced lipid availability during erythroid culture and offer new insights into how culture media affects the development and functionality of lab grown blood.

**Supplementary Information:**

The online version contains supplementary material available at 10.1038/s41598-026-37229-z.

## Introduction

Red blood cells (RBCs) are continuously subjected to shear stress during circulation and have to deform repeatedly to traverse narrow capillaries and splenic slits^1^. This exceptional deformability is largely due to their highly specialised membrane, which comprises a spectrin based cytoskeleton anchored to a cholesterol rich lipid bilayer. Unlike most cellular membranes which typically contain 10–30% cholesterol, the RBC membranes contain up to 50% cholesterol out of total membrane lipids^2^, a feature critical for maintaining mechanical stability, fluidity and overall function.

The connection between cholesterol and erythrocyte physiology has been recognised for over a century since cholesterol was first identified in red cell membranes. Studies have since shown cholesterol levels influence RBC deformability^3,4^ and are linked to haematological disorders associated with altered blood viscosity^5^. Dysregulation of cholesterol homeostasis affects RBC lifespan, clearance, and oxygen transport efficiency^5–7^. For example, foetal erythropoiesis involves up-regulation of cholesterol metabolism^8^, and is linked to activation of p53-dependent activation of cholesterol uptake by ABCA1 and suppression of cholesterol synthesis via the mevalonate pathway^9^. This regulatory axis is essential during adult erythropoiesis, where p53 influences iron uptake by controlling the transcription of the ferrireductase Steap3^10^. Hypercholesterolemia has also been reported as disrupting RBC functional and structural properties, by accumulating cholesterol within the plasma membrane cell deformability is reduced, impacting oxygen transport^11^. In mature RBCs, cholesterol depletion, which occurs during refrigerated storage, has been linked to changes in morphology, increased membrane rigidity^12^, reduced deformability and, enhanced splenic clearance^13^. These findings highlight the importance of maintaining cholesterol availability during in vitro erythroid culture, where lab-grown reticulocytes must emulate native RBCs membrane properties to survive and function effectively in vivo.

The heightened interest in lab-grown RBCs as potential blood substitutes has intensified the focus on the characteristics of cultured reticulocytes and how closely they mimic their native counterparts. Previous studies have shown that lab-grown reticulocytes exhibit similar blood group antigen expression to their originating donor cells^14,15^, possess a proteomic profile comparable to native reticulocytes^16,17^, and are capable of deforming^18,19^. Furthermore, genetic modifications introduced into cultured erythroid cells to mimic patient specific mutations yield reticulocytes that display the characteristic phenotype expected, provided the phenotype manifests at the immature RBC stage^20–22^. These findings suggest that lab-grown reticulocytes possess requisite membrane protein structures and interactions, supporting their potential as functional RBC substitutes.

One currently underexplored factor influencing the properties of lab grown blood is their plasma membrane lipid composition. Outside the highly regulated and nurturing in vivo bone marrow microenvironment, the impact of exogenously supplied lipid sources used during erythroid culture is of particular importance. In the RESTORE clinical trial (https://www.isrctn.com/ISRCTN42886452*)*, which is currently evaluating the performance of lab-grown reticulocytes compared to donor derived red blood cells in healthy volunteers, AB serum (alongside human serum albumin (HSA)) is used as a lipid source and no detrimental effects observed on the characteristics of the lab-grown reticulocytes. In contrast, several studies^19,23,24^ have reported cholesterol deficiencies in cultured reticulocytes, which required supplementation to preserve reticulocyte integrity. These discrepancies are often difficult to interpret due to inherent variability in experimental reagents, donor cell sources and culture protocols. Nevertheless, we hypothesised that the observed differences may be attributable to the lipid sources employed- particularly as the latter studies all used (S/D)-extracted plasma. Addressing this question requires a more standardised and systematic comparison of lipid supplements used in erythroid culture systems.

In order to systematically assess the lipid content of cultured reticulocytes produced using distinct lipid sources, lipids were provided through supplementation of *in vitro* erythroid cultures with AB serum, or S/D-extracted plasma in the presence or absence of cholesterol supplementation. Our findings demonstrate that the choice of lipid sources in erythroid culture significantly affects cholesterol availability, which in turn affects cell yield and reticulocyte characteristics. Moreover, we provide the first comprehensive lipidomic, metabolomic, and proteomic comparisons of reticulocytes derived from these diverse lipid sources.

## Results

### Reticulocyte yield is impacted by choice of lipid source in differentiation media

**Fig. 1:**
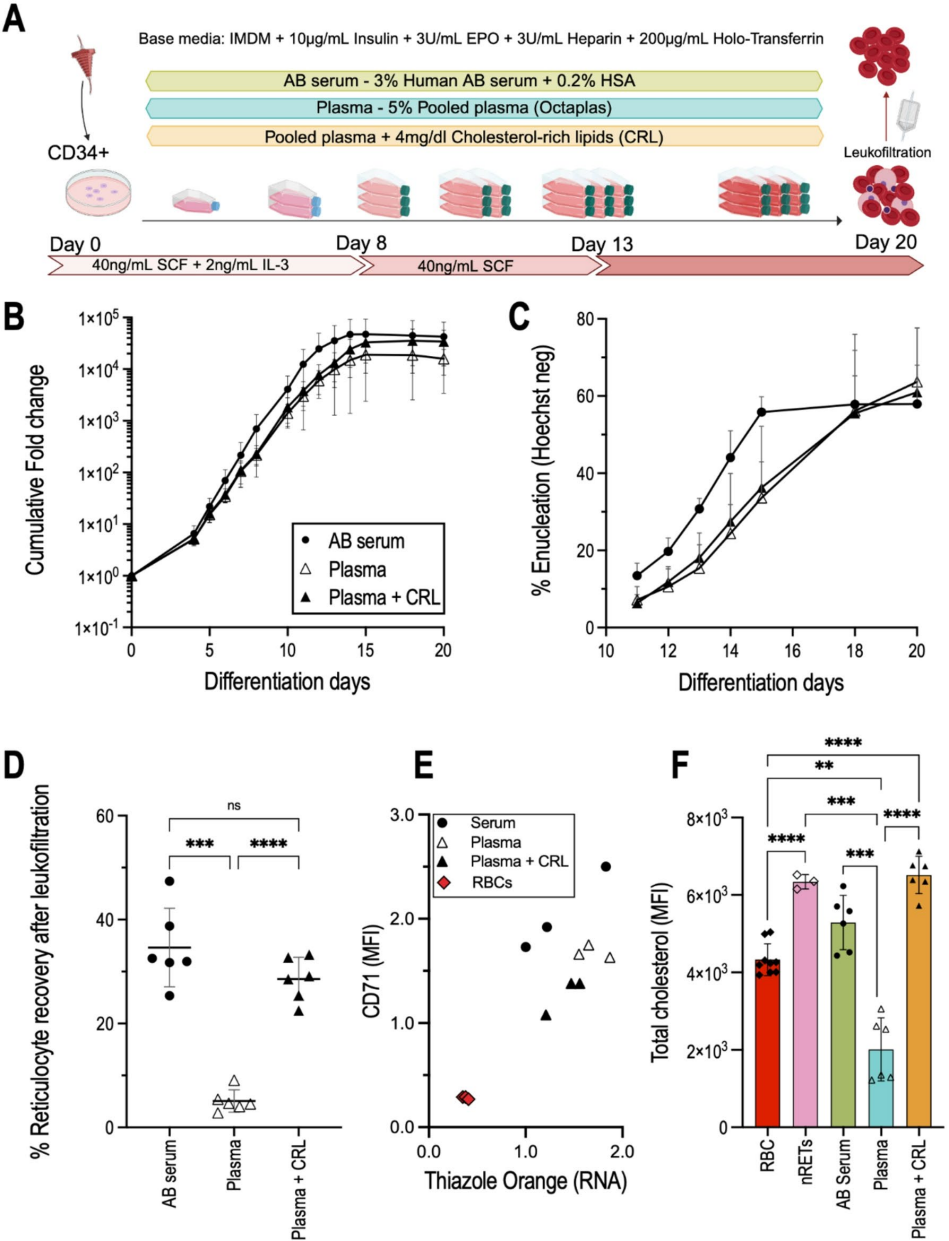
Lipid source used in primary *in vitro* erythropoiesis affects cell expansion, enucleation rate, and filtration yields. (**A**) CD34-positive cells (human hematopoietic stem cell marker) are isolated from leukocyte reduction filters and cultured for 20 days using a three-step culture protocol. The base media consists of Iscove’s Modified Dulbecco’s Medium (IMDM) supplemented with insulin, erythropoietin (EPO), heparin, holo-transferrin, and either AB serum or Plasma as lipid sources. The addition of cholesterol-rich lipids (CRL) to plasma cultures was also explored. In the first stage, days 0–7, media is supplemented with stem-cell factor (SCF) and interleukin-3 (IL-3). The second stage until day 13 depends only on SCF supplementation and the third being only base media. On day 20 cells are leukofiltered to obtain a pure reticulocyte population. (**B**) Cumulative fold expansion (log_10_ scale) of adult CD34 + cells differentiation grown in media containing either human AB serum, pooled human plasma, or pooled plasma supplemented with Cholesterol-Rich-lipids (CRL). (**C**) Percentage enucleation from day 11 of differentiation. Reticulocytes were identified by Hoechst negativity. *n* = 6, error bars represent the standard error of the mean. (**D**) Percentage of reticulocytes recovered after leukofiltration on day 20 of erythroid differentiation. A parametric (normality confirmed with a Shapiro-Wilk test) Brown-Forsythe and Welch test was performed to test for differences between groups. *p* < 0.05 was considered statistically significant. *n* = 6, error bars represent the standard deviation. (**E**) Median fluorescent intensity of CD71 (transferrin receptor) plotted against Thiazole Orange (RNA content) of filtered reticulocytes. Individual values for each donor (*n* = 3) are represented, as well as three control red blood cell samples. Shaded regions correspond to a 95% confidence level calculated using a linear regression model in Rstudio. (**F**) Total cholesterol of matched samples (to panel **A**) was measured by flow cytometry using a filipin stain to associate osmotic fragility with cholesterol levels. The values were normalised to the RBCs. A parametric Brown-Forsythe and Welch test was performed to test for significance.

To elucidate the role of lipid supplementation in *in vitro* erythroid cultures, multiple lipid sources were tested in adult hematopoietic stem cell (HSC) cultures. We used a well-established three-phase culture method comprised of Iscove’s Modified Dulbecco’s Medium (IMDM) supplemented with erythropoietin (EPO), insulin, heparin, holo-transferrin, as base media^25^. The culture media composition was kept identical but the lipid sources used were either 3% AB serum and 2% HSA (called here AB serum) or 5% human pooled plasma (brand name Octaplas^®^, referred to as Plasma for simplicity), or plasma supplemented with 50 mg/L cholesterol-rich-lipids (Plasma + CRL). Figure 1A summarises the culture conditions tested, and the culture protocol followed. Cells were cultured for 20 days with Fig. 1B illustrating the cumulative fold expansion of the erythroid cultures in the three tested media compositions. The standard deviation between the 6 donors tested is high, particularly when cultured in AB serum, most likely due to donor biological variability in terms of progenitor expansion. The AB serum cultured cells tend to experience a higher proliferation from day 5 onwards compared to the plasma culture, plateauing at 4.8±4.5 × 10^4^-fold at day 15. Plasma-cultured cells on average exhibited only 1.9±1.1 × 10^4^-fold proliferation on day 15 and the supplementation with CRL increased proliferation to 3.6±2.4 × 10^4^-fold on day 18.

From days 11 to 20, enucleation was assessed on the basis of Hoechst negativity (Fig. 1C). Until day 18 the percentage of reticulocytes in the AB serum culture is greater than both Plasma cultures, indicating a faster progression through differentiation. Values remain constant for the AB serum cultures day 15 onwards, contrary to Plasma which show a steady increase until day 18. Despite these differences, all three conditions yielded similar enucleation values at the time of harvest of the culture, an average of 61.0% ±2.9% (standard deviation, SD). Notably, supplementation with cholesterol does not alter enucleation percentages. For the same culture media composition containing AB serum, values between 10^4^ and > 10^5^-fold expansion and > 60% enucleation have been reported^25,26^, consistent with the data collected here.

At the end of differentiation, the culture consists of a mixture of reticulocytes, pyrenocytes, and nucleated cells (mostly orthochromatic erythroblasts). To obtain a pure sample of reticulocytes (> 98%) leukocyte reduction filters can be used to isolate the reticulocytes. Leukofilters are normally used after whole blood donations to eliminate white blood cells prior to transfusion, relying on the deformability of RBCs to easily pass through the filter while the stiffer nucleated cells are retained^27^. The cultures were filtered on day 20 and the yield (Fig. 1D) was calculated based on total pre- and post-filtration cell counts. After filtration, the AB serum culture condition averaged a yield of 34.6 ±7.5% (SD) while Plasma-cultured cells only achieved 5.1 ±2.2% reticulocyte recovery, a significant difference highlighting the decreased deformability of reticulocytes grown in Plasma. The addition of cholesterol-rich lipids to the plasma containing media rescued filterability levels to 28.9 ±4.1%.

The degree of reticulocyte maturation of the filtered cultures was next assessed through flow cytometry (Fig. 1E) by plotting the expression of the transferrin receptor (CD71), a marker absent on mature erythrocytes, against Thiazole Orange – measuring residual RNA^28^. The individual data points indicate a higher CD71 expression, as judged by comparing MFI on AB serum derived reticulocytes (RET^AB serum^), compared with Plasma-derived (RET^Plasma^), either with or without cholesterol supplementation (RET^Plasma+CRL^).

Next, total cholesterol was assessed by Filipin stain (Fig. 1F). This indicated a significant increase in total cholesterol of native RETs (nRETs) and RET^AB serum^ compared with RBCs. RET^Plasma^ had a 54% decrease in total cholesterol when compared to RBCs (2.0±0.8 × 10^3^ against 4.3±0.4 × 10^3^), and 68% when compared to nRET (6.3±0.2 × 10^3^). It should be noted that these results were obtained on the filtered reticulocytes, which only accounted for 5% of total reticulocytes present pre-filtration for plasma cultured cells. Also, although we use extensive washing to remove excess cholesterol micelles, we highlight filipin staining reflects total cholesterol rather than exclusively membrane incorporated cholesterol. Upon cholesterol supplementation of plasma-cultured reticulocytes, total cholesterol levels increased to levels similar to that observed in nRETs. In fact, there is no significant difference between nRET, RET^AB serum^, and RET^Plasma+CRL^, as all were higher than RBCs, although not significantly for RET^AB serum^.

### Plasma grown erythroid cells have compensatory changes consistent with exposure to low cholesterol levels

**Fig. 2:**
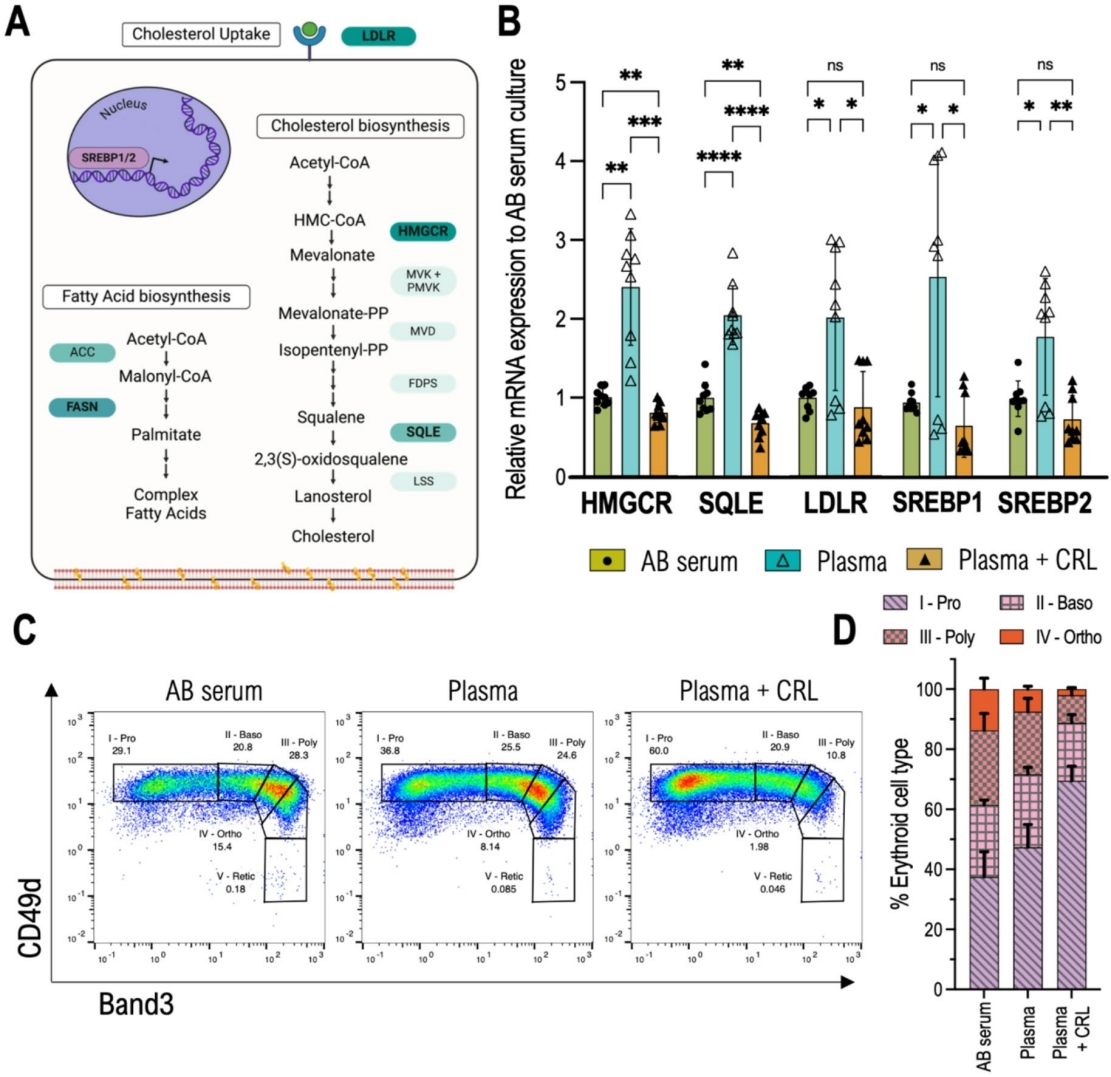
qPCR shows cholesterol biosynthesis is significantly increased in plasma cultured cells. (**A**) Main steps in cholesterol and fatty acid biosynthesis. Cholesterol biosynthesis begins with acetyl-CoA and proceeds via HMG-CoA reductase (HMGCR), forming mevalonate and downstream intermediates (mevalonate-PP, isopentenyl-PP, and squalene). Squalene epoxidase (SQLE) converts squalene to 2,3(S)-oxidosqualene, leading to lanosterol and cholesterol synthesis. Fatty acid biosynthesis also originates from acetyl-CoA, with acetyl-CoA carboxylase (ACC) producing malonyl-CoA, followed by fatty acid synthase (FASN) generating palmitate and complex fatty acids. SREBP1/2 transcription factors regulate these pathways, with SREBP1 driving fatty acid synthesis and SREBP2 controlling cholesterol biosynthesis. The LDL receptor (LDLR) mediates cholesterol uptake, maintaining lipid homeostasis. Made using Biorender. (**B**) mRNA levels of HMGCR (HMG-CoA reductase), SQLE (Squalene Epoxidase), LDLR (Low density lipoprotein receptor), SREBP1 and SREBP2 (Sterol regulatory element-binding protein 1 or 2) relative to GAPDH on day 8 of differentiation after CD34 + isolation. A parametric Brown-Forsythe and Welch test was performed to test for differences between groups. *p* < 0.05 was considered statistically significant (*n* = 3, *N* = 3). (**C**) Example waterfall plots of cells the same donor on day 8 of erythroid differentiation, labelled against Band3 and CD49d, grown in medium containing AB serum, Plasma, or Plasma supplemented with CRL. Corresponding percentage of each stage (I-Proerythoblast, II-Basophilic, III-Polychromatic, IV-Orthochromatic, and V-Reticulocyte) is indicated next to the corresponding gate. D Shows percentage of erythroid cell types present on day 8 for each condition studied, quantified as displayed in panel above (*n* = 3). Error bars represent the standard deviation.

Expression levels of key genes important for cholesterol homeostasis were evaluated using real-time quantitative PCR on RNA isolated from erythroblasts exposed to different lipid sources at day 8 of differentiation. Multiple genes known to control cholesterol biosynthesis were evaluated (Fig. 2A) including the sterol-regulatory element-binding proteins 1 and 2 (*SREBP1* and *SREBP2*) expression (a transcription factor that controls cholesterol and lipid homeostasis), alongside 3-hydroxy-3-methylglutaryl-coenzyme A reductase (*HMGCR*) and Squalene monooxygenase (*SQLE*) which are both rate limiting enzymes in the cholesterol biosynthetic pathway. We also evaluated low-density lipoprotein receptor (*LDRL*) expression which plays a crucial role in cholesterol uptake.

Gene expression was significantly increased in the Plasma condition for all genes analysed (Fig. 2B) and was either similar or reduced upon CRL supplementation when compared to AB serum. *HMGCR*, *SQLE*, *LDLR*, and *SREBP1* all show greater than 2-fold increase in expression (2.4 ± 0.74, 2.1 ± 0.38, and 2.0 ± 0.94, 2.7 ± 1.6 respectively). *SREBP2* had a 1.8 ± 0.76 increase in Plasma culture compared with AB serum. However, it is notable one donor exhibited a less pronounced response, for all genes except SQLE, to the low cholesterol environment, suggesting that that there may be inherent variability among donors. When CRL supplementation occurs alongside plasma, all genes have a mean fold-expansion inferior to 1 in the Plasma + CRL condition, indicating lower gene expression than when cultured in AB serum.

To control for possible differences in gene expression associated with differentiation progression the different cell stages of erythropoiesis were assessed by observing the expression of membrane proteins Band 3 and CD49d, with Band 3 increasing during terminal differentiation while CD49d decreases^29^. Using one donor as an example, Fig. 2C exemplifies the gating strategy used to determine the percentages of erythroid cell type (Proerythroblast (I), Basophilic Erythroblast (II), Polychromatic Erythroblast (III), and Orthochromatic Erythroblast (IV), Reticulocyte (V)) for tested conditions. The combined results for 3 donors are visible in Fig. 2D.

Interestingly, Fig. 2D shows that both plasma-containing conditions differentiated slower than AB serum, with the latter having only 37.5 ± 8.31% of total cells left as Proerythroblasts while Plasma-only had 47.3 ± 7.57%, and Plasma + CRL 69.5 ± 4.82%. This observation suggests that the restoration of gene expression levels observed in the Plasma + CRL cells to those comparable with AB serum is not due to similarities in erythroid cell populations but rather a response to cholesterol availability.

### Proteomics, metabolomics and lipidomic studies

To more extensively explore the effects of media composition on the reticulocytes produced from the different lipid sources, samples of filtered reticulocytes and RBCs were subjected to multi-omics analysis.

**Fig. 3:**
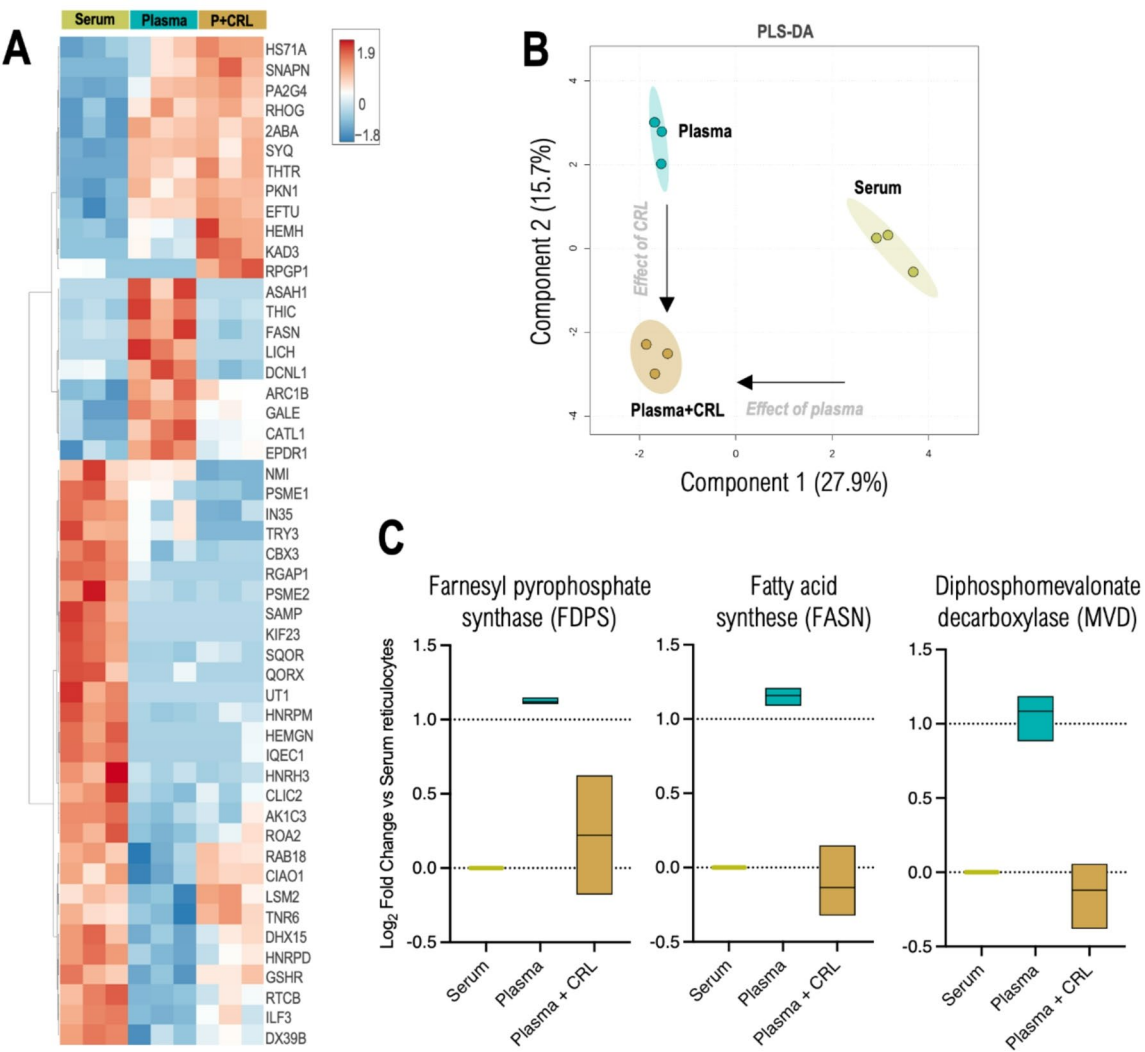
Summary of proteomic differences in CD34^+^ cell-derived reticulocytes grown in the presence of Serum, Plasma, or Plasma supplemented with cholesterol-rich lipids. Serum, Plasma and Plasma + CRL (P + CRL) compared with control RBCs (top 50 proteins by ANOVA in **A** and Partial least square-discriminant analysis – PLS-DA in **B**). (**C**) Box plots of upregulated proteins found in Plasma treated samples when compared with donor matched Serum. The log_2_(FC) was analysed for significance with non-parametric Kruskal-Wallis test with Dunn’s correction (*p* < 0.05).

The proteomic dataset was analysed initially by normalising the Max Label Free Quantification values against donor-matched RBCs (Fig. 3A,B). Many of the proteins highlighted where abundance significantly changes across the three conditions were associated with processes related to reticulocyte maturation and red cell-specific functionality, including membrane trafficking, carbon dioxide transport, and hydration regulation. Although, notably, Fatty acid lipid synthase (FASN) was detected as upregulated in the plasma culture reticulocytes. To more precisely dissect the differences induced by the extracellular environment, we performed a second analysis normalising instead to AB serum-cultured reticulocytes, allowing for the quantification of 1021 proteins, up from 500 when comparing against RBCs, effectively doubling the proteomic depth. This aligns with findings from Gautier et al.^30^ who identified 654 proteins as being reticulocyte specific. The Enrichr software tool was used to detect functional relationships between the protein groups. Of note was the identification of three proteins significantly upregulated in Plasma only-grown reticulocytes, observed in Fig. 3C – Farnesyl pyrophosphate synthase (FDPS), FASN, and Diphosphomevalonate decarboxylase (MVN), all known to be involved in lipid and cholesterol biosynthesis. All three proteins are regulated by the transcription factors SREBP (Reactome pathway knowledgebase 2022, *p* = 0.00002441), found to also be upregulated in the differentiating plasma-grown erythroid cells by RT-qPCR (Fig. 2B).

Metabolomics data was next analysed by comparing metabolites in Plasma or Plasma + CRL grown reticulocytes to AB serum reticulocytes. We observed that most metabolites (*Supplementary Fig. 1*) have no significant alteration between culture systems and, even if observed to be significant these often translated into modest changes associated with a large degree of variability observed across the 3 donors analysed. Of note was the detected upregulation in UMP levels, particularly in the presence of cholesterol supplementation, and a reduction in pyridoxal. Decreases in succinate and citrate highlight alterations in the TCA cycle. The depletion of gamma-L-glutamyl-L-cysteine (precursor to glutathione^31^ and the increase in L-selenomethionine (common intermediate used for synthesising selenocysteine, essential for peroxidase activity^32^ also indicate alterations in antioxidant response.

**Fig. 4:**
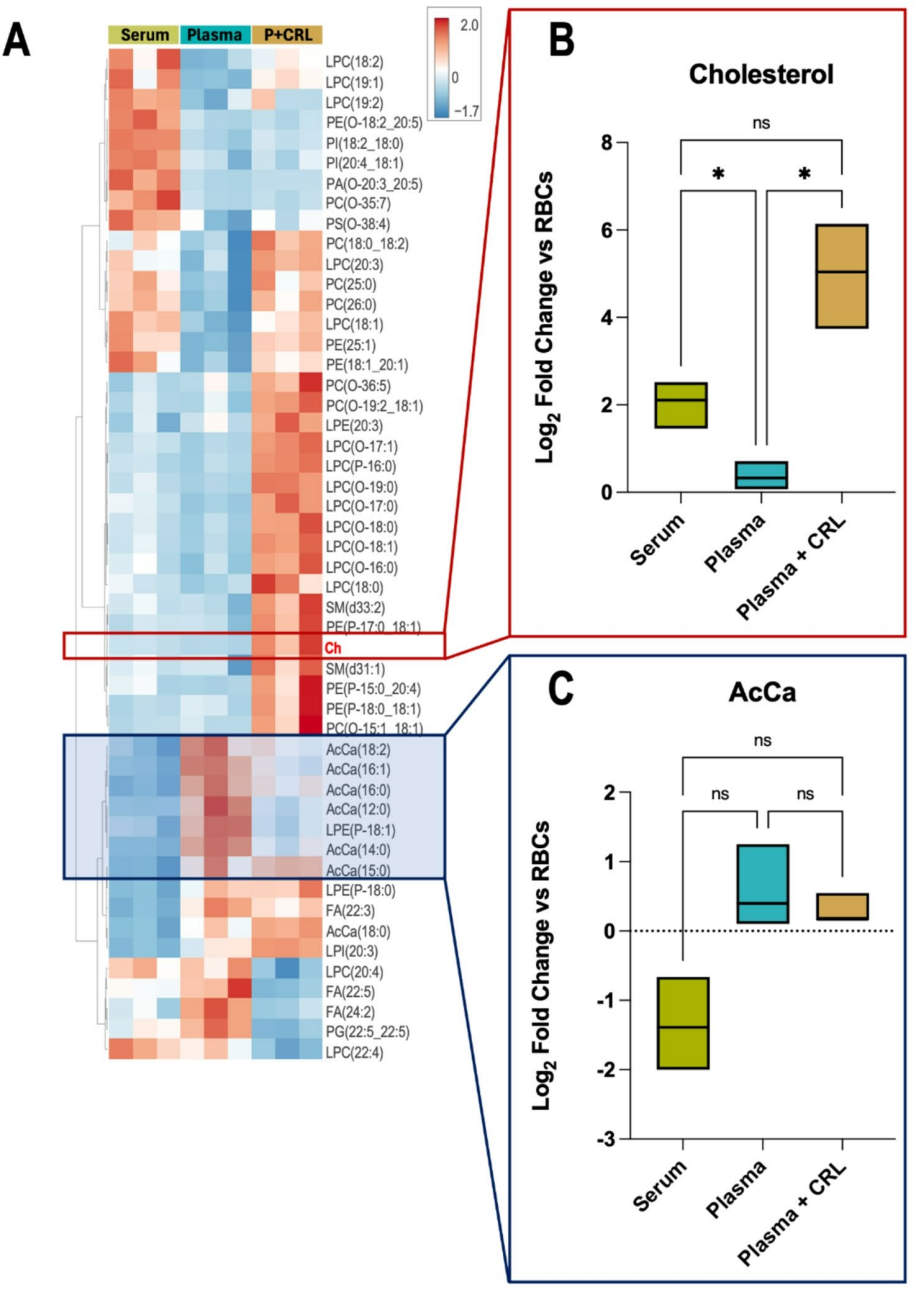
Lipidomic analysis confirms increased cholesterol in serum and supplemented plasma grown primary reticulocytes. (**A**) Summary heat map of the top 50 significant lipid changes by ANOVA of the lipidome data from serum, plasma, and plasma + CRL cultured reticulocytes in comparison with donor matched erythrocytes. The values represent the average of log_2_(Fold change, FC) of the sum of peak areas of each chain against the equivalent sum in matched-donor red cells. (**B**,**C**) Box plots of Cholesterol (Ch, **B**) and acyl-carnitines (AcCa, **C**) log_2_(FC) analysed for significance with non-parametric Kruskal-Wallis test with Dunn’s correction (*p* < 0.05).

A comprehensive lipidomics comparison was also conducted, and a summary heat map of the top 50 significant lipid changes identified between the cultured reticulocytes and matched RBCs is shown in Fig. 4A. This identified several lipid classes with similar or higher abundance compared with red blood cells, which aligns with the fact that cultured reticulocytes must still lose around 20%^33^ of their membrane surface area during the maturation process. Importantly, cholesterol levels were reduced in plasma, (Chol, Fig. 4B) whereas cholesterol levels were observed to be significantly increased in both serum grown and Plasma supplemented with CRL reticulocytes relative to erythrocytes. Acylcarnitines (AcCa, Fig. 4C) were instead found to be less abundant in serum-grown reticulocytes compared with both Plasma conditions, although this difference is not significant.

### Reduced cholesterol in the plasma-grown reticulocytes impacts reticulocyte characteristics

**Fig. 5:**
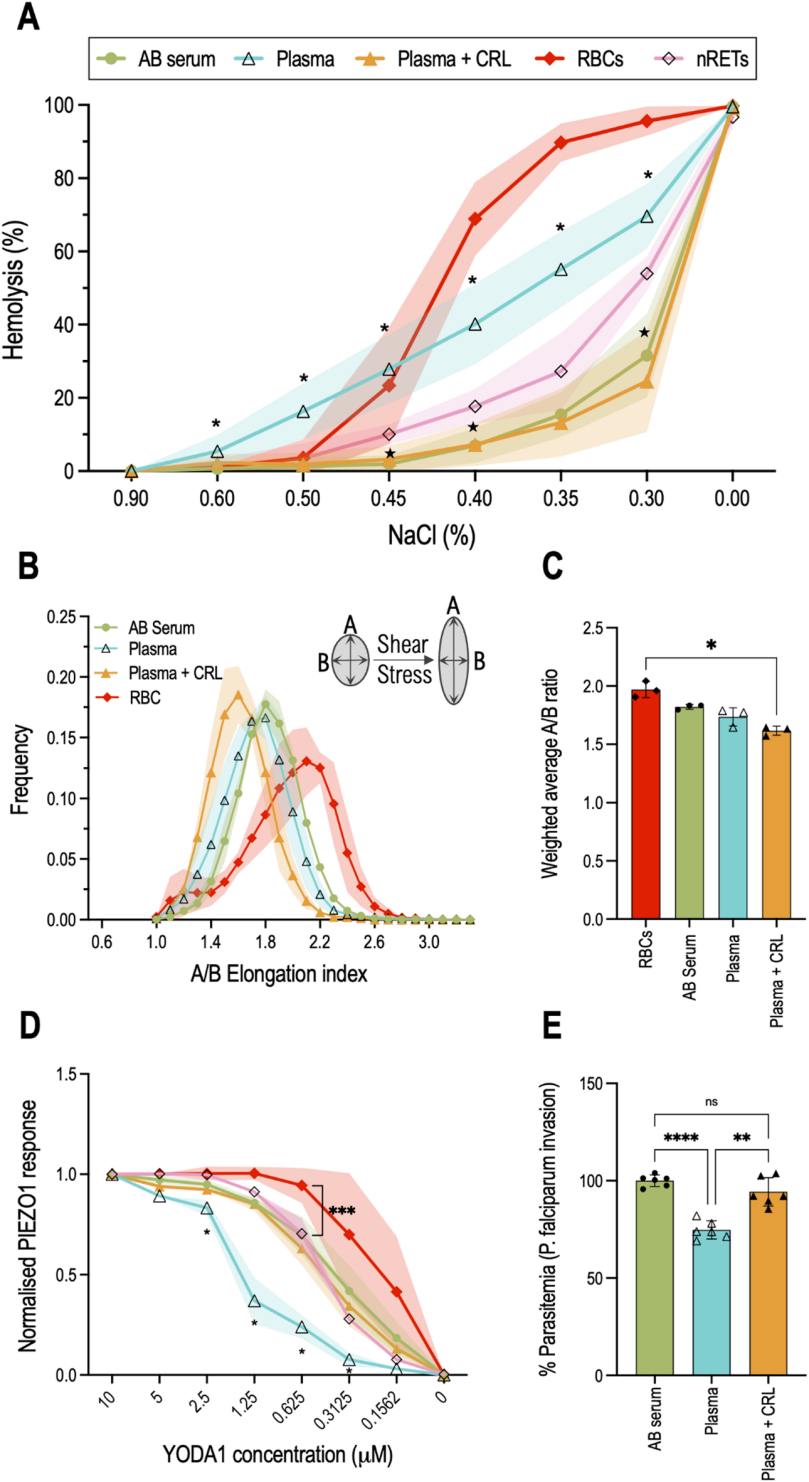
Cholesterol deficiency in cultured reticulocytes modulates deformability, Piezo1 activity and *P. falciparum* invasion. (**A**) Osmotic fragility analysis of red blood cells (RBCs, *n* = 9), native reticulocytes (nRETs, *n* = 3, CD71+) and filtered reticulocytes cultured in media containing either AB serum (*n* = 6), Plasma (*n* = 6), or Plasma supplemented with Cholesterol-Rich-lipids (*n* = 6). For each donor (n) two technical replicates were performed. Osmotic resistance was calculated based on viable cell counts (flow cytometer) after incubation with decreasing concentrations of NaCl. Half-maximal haemolysis (IC_50_) values with 95% confidence intervals were determined using a variable-slope sigmoidal fit (see Supplementary Fig. 3). Non-parametric Mann-Whitney U test with subsequent Dunn correction was performed to test for differences between groups. ∗ *p* < 0.05 Plasma versus nRETs; ★ *p* < 0.05 AB serum versus nRETs. (**B**) Deformability of RBC and cultured reticulocytes measured under shear stress by Automated Rheoscope Cell Analyser, which elongates cells and measures length over width as deformability parameter. (*n* = 3 for each conditions, min of 5000 cells analysed for each). (**C**) Weighted average of A/B elongation index for each condition (*n* = 3). Statistical significance was assessed by Kruskal-Wallis test with Dunn’s correction. (**D**) Piezo1 activity of RBCs (*n* = 6), nRETs (*n* = 3), and filtered reticulocytes (*n* = 3 each culture condition). Calcium concentration inside the cell is measured pre- and post-YODA1 addition, a chemical inducer of Piezo1 activity, correlating with Piezo1 activity. Data shown is normalised for the highest YODA1 concentration used, 10µM. Multiple t-tests with Welch’s and Holm-Šidak corrections were performed to test for differences between groups. * signifies a *p* < 0.05 for Plasma versus nRETs and *** equals to *p* < 0.001. (**E**) Bar plot quantifying *P. falciparum* reticulocyte invasion efficiency. Invasion was assessed by flow cytometry using a SYBR-green DNA stain (2 separate parasitaemia percentages) and data was normalised to the NT-control (*n* = 3, SD). A parametric Brown-Forsythe and Welch test was performed to test for differences between groups. Plotted data corresponds to the mean with error bars or shaded regions indicating standard deviation. *p* < 0.05 was considered statistically significant.

As reported previously^23,35,36^, an osmotic fragility test (OFT) can be used to assess reticulocyte fragility. The OFT exposes cells to various dilutions of NaCl, exposing RBCs to a hypotonic environment, causing water to enter the cell, resulting in swelling and ultimately lysis. The OFT was performed on RBCs, native reticulocytes (nRETs) obtained by magnetically isolating CD71^+^ cells from donor red cells and filtered cultured reticulocytes. As shown in Fig. 5A, RBCs produce an S-shape curve consistent with 50% haemolysis observed between 0.45% and 0.40% NaCl (w/v) whilst native reticulocytes are more resistant to osmotic changes, requiring a concentration of 0.35% to 0.30% NaCl (w/v) to achieve the same cell lysis percentage. This is consistent with the consensus that reticulocytes are more resistant to osmotic alteration despite being less deformable than RBCs^37^. Both RET^AB serum^ and RET^Plasma+CRL^ reticulocytes exhibit similar OFT pattern to that observed for nRETs but with significant differences at 0.45%, 0.40% and 0.30% NaCl solutions (w/v). These data indicate a heightened resistance to hypotonic solutions of cultured reticulocytes when compared with nRETs. Plasma-grown reticulocytes display the opposite, an increased fragility even when exposed to high salt concentrations. At 0.5% NaCl (w/v) RET^Plasma^ experience 16.3 ±7.6% haemolysis while only 3.4 ±4.1% of nRETs and 1.2 ±2.4% of RET^AB serum^ lyse at that same NaCl concentration. This remains a significant difference throughout the remaining NaCl dilutions until all conditions 100% lyse in water.

To assess the properties of the reticulocytes produced using the different lipid sources, reticulocyte deformability was measured by Automated Rheoscopy (ARCA)^34^, a single cell ektacytometry system where cells are subjected to a constant and specific shear stress field causing cell elongation. The A/B ratio is used as a deformability measurement, plotted against the frequency of said ratio in the cell population (Fig. 5B). The weighted average of the A/B ratio was calculated for all 3 donors and the values were plotted in Fig. 5C. Red blood cells achieve on average a higher A/B ratio, at 1.96 ±0.06 (SD), followed by RET^AB serum^ with 1.82 ±0.02 (SD), RET^Plasma^ at 1.73 ±0.08 (SD) and RET^Plama+CRL^ at 1.61 ±0.04 (SD). The higher elongation index observed in RBCs compared to cultured reticulocytes is in line with the well-established understanding that mature RBCs are more deformable. Statistically, there is no difference between the means of the three reticulocyte samples but when analysing the distribution peaks in Fig. 5B there is a visible shift of RET^Plasma+CRL^, indicative of reduced deformability. In fact, comparing the A/B ratio average of cultured reticulocytes with RBCs, only RET^Plasma+CRL^ is substantially reduced, once again indicating a decrease in deformability correlated with the increased cholesterol content.

Multiple reports have highlighted the importance of the lipid environment on the mechanosensory channel Piezo1 properties and activity^38^, as such alteration in the different lipid sources and the resulting cholesterol changes could impact Piezo1 function. In order to investigate this RBCs, nRETs, and filtered cultured reticulocytes cultured in the different lipid sources were treated with various concentrations of YODA1. A baseline is first acquired for each sample followed by YODA1 addition which results in a Ca^2+^ influx, detected by Fluo4-AM fluorescence. The YODA1 titration results are plotted in Fig. 5D, normalised for the highest concentration used (10µM).

Between 10µM and 1.25µM YODA1, no discernible difference in Ca^2+^ influx is observed in erythrocytes, indicating saturating activation of Piezo1. This saturation is visually evident through the plateau in Piezo1 response. The channel activation experiences a gradual decline with decreasing YODA1 concentrations. Comparing nRETs with RBCs there was a difference in response from 1.25µM, with RBCs consistently exhibiting higher Ca^2+^ influxes, also obvious by the required concentration to achieve 50% Piezo1 activation (approximately 0.20µM to 0.46µM). As mentioned before this is to be expected, as reticulocytes are less deformable. Focusing on cultured reticulocytes, there is no difference between nRETs and RET^AB serum^, indicating that the differences observed between cultured reticulocytes and RBCs are due to their immaturity and not from *ex vivo* erythropoiesis. There is however a detectable decrease of Piezo1 responsivity observed for RET^Plasma^, that was recovered in full by cholesterol supplementation, as RET^Plasma+CRL^ display the same channel activation levels as nRETs.

Finally, cholesterol also plays a pivotal role in *Plasmodium falciparum* invasion of RBCs as the parasite relies on cholesterol-rich domains in the host cell membrane as entry points. Disruption of cholesterol homeostasis has been shown to impede the invasion process^39^. Figure 5E illustrates invasive susceptibility of all three culture conditions (AB serum, Plasma, Plasma + CRL), with invasions assays performed on filtered reticulocytes and data normalised for RET^AB serum^. Consistent with the lower cholesterol levels present in RET^Plasma^, a significant reduction in invasion capacity was observed for these reticulocytes. This effect is rescued by cholesterol addition in the RET^Plasma+CRL^ cultured cells.

## Discussion

This study has confirmed that the lipid profile of reticulocytes produced *in vitro* by culturing primary stem cells (CD34^+^) is reassuringly similar to the lipid profiles of native reticulocytes and erythrocytes. It has also confirmed that a significant factor that can influence reticulocyte quality and functionality is the choice of lipid supplementation, particularly the use of human AB serum versus solvent-treated plasma products such as Octaplas. Our findings show that when using S/D-extracted plasma such as Octaplas, careful optimisation of cholesterol supplementation should be undertaken to ensure that the reticulocytes do not have cholesterol deficiency and so impact on their characteristics. We also show that supplementation of cultures with 3% AB serum provide sufficient cholesterol for erythroid differentiation. Consequently, additional cholesterol supplementation is not required when AB serum is used. These findings underscore the broader challenge in erythroid culture standardisation, as different laboratories employ varied serum, plasma, or serum-free media formulations, each method will require careful optimisation, characterisation and testing to check for impact on reticulocyte characteristics and functionality.

Despite the challenge of donor biological variability in terms of proliferation, it was noticeable that under the culture conditions using AB serum as a lipid source may yield better results than plasma alone in terms of proliferation (Fig. 1B), with cholesterol supplementation further enhancing the proliferation of plasma-grown cells. Given the rapid proliferation of early erythroid progenitors, it is expected that cholesterol availability would impact cell expansion^19,40^. Co-labelling with Band 3 and CD49d (Fig. 2C) corroborated data observed regarding enucleation (Fig. 1C), where reticulocytes cultured in plasma progressed slower through differentiation. This was independent of cholesterol availability, as CRL supplementation of Plasma cultures did not reverse this effect, marking a key difference between Plasma and Serum. A similar effect was observed regarding CD71 levels, a marker for reticulocyte maturity, with plasma reticulocytes showing reduced expression levels. A curious result given the apparent delayed differentiation observed in the early stages of culture (Fig. 1E).

Importantly, plasma only cultures exhibit dramatically impaired filtration, producing extremely low recovery yields following leukofiltration (Fig. 1D). Adding cholesterol-rich lipids to the plasma containing media rescued the reticulocyte filterability to levels observed in AB serum cultures. This is suggestive of impacted deformability, as successful passage through the leukofilter is highly dependent on cell deformability^27^. A recent study by Claessen et al.^19^ explored the requirement for cholesterol supplementation as an aid to improved filterability and in their study, using plasma as a lipid source. Of note here is the novel correlation between the specific use of plasma in culture media and insufficient cholesterol availability, since erythroid differentiation in the presence of AB serum does not require cholesterol supplementation. This was corroborated by measuring cholesterol levels in the filtered reticulocytes (Fig. 1F), which revealed reduced cholesterol in RET^Plasma^ compared with even mature RBCs and is also observed in the comparative lipidomics.

The observed upregulation of *HMGCR*, *SQLE*, and *LDLR* genes in erythroid cells grown in plasma-only conditions is consistent with the hypothesis that the cultured erythroblasts are experiencing cholesterol restriction and so are compensating for the lower cholesterol availability by increasing cellular cholesterol synthesis and uptake. The downregulation of the same genes when cholesterol is supplemented in the culture media is consistent with a feedback mechanism where exogenous cholesterol inhibits the need for intracellular synthesis and uptake. This feedback control is well documented in cholesterol metabolism, where excess cholesterol leads to suppression of biosynthesis and receptor-mediated uptake to maintain homeostasis^41,42^.

SREBP2 mRNA levels also increase in response to low cholesterol conditions, further amplifying the regulatory network that drives the expression of key cholesterol biosynthesis and uptake genes such as HMGCR, SQLE, and LDLR. Interestingly, the observed upregulation of SREBP2 in plasma-only conditions was quite modest, showing less than a two-fold increase, with significant variation between donors. Indeed one donor exhibited no upregulation of the transcription factor and correspondingly low expression of regulated genes HMGCR, SQLE, and LDLR, while the remaining two exhibited more substantial regulation of these genes.

SREBP1 was also significantly upregulated in the same two donors, suggesting that fatty acid metabolism pathways can also be activated alongside cholesterol biosynthesis. In fact, despite SREBP1 being more associated with transcription of fatty acid synthesis genes and SREBP2 more associated with cholesterol-related genes, recent publications have shown how SREBP1 is also activated by low cholesterol and how the upregulation of FASN (Fatty Acid Synthase), a downstream target of SREBP1, has been linked to enhanced cholesterol production^43–46^. Our proteomics analysis of filtered reticulocytes further corroborates this observation, detecting a marked upregulation of FASN in Plasma-cultured cells only. Additionally, the upregulation of FDPS (Farnesyl Diphosphate Synthase) and MVD (Mevalonate Diphosphate Decarboxylase) in the proteomics data indicates an increased flux through the mevalonate pathway, crucial for cholesterol biosynthesis^47^.

Overall, the omics data showed that plasma-grown reticulocytes exhibit a marked activation of lipid and cholesterol biosynthesis pathways, likely driven by SREBP1/2 activity, fuelled by reduced cholesterol depletion. These data also reveal minimal proteomic difference between cultured reticulocytes and mature red blood cells, independent of lipid source used.

We established that RET^AB serum^ have similar osmotic resistance profile to those of nRETs, whilst RET^Plasma^ showed increased osmotic fragility (Fig. 5A), consistent with previously published data of cholesterol-deficient reticulocytes levels^23^. The osmotic fragility was reversed by supplementation with CRL. Curiously when submitting the same reticulocytes to an automated rheoscope (Fig. 5B-C), testing the elongation capacity of the cell, we saw no impairment of the plasma cultured cells, instead the reticulocytes supplemented with extra cholesterol had reduced deformability which was anticipated, because increased membrane stiffness has been described in hypercholesterolemia^7,48^, aligning with our findings. This suggests that careful optimisation of cholesterol supplementation should be considered, to ensure minimal impact on reticulocyte properties.

Cholesterol not only influences membrane fluidity and stiffness but also regulates the activity of membrane proteins, such as the mechanosensitive ion channel PIEZO1. Studies have shown that PIEZO1 function and activity are dependent on cholesterol-rich lipid rafts^49,50^. By employing a titration of the PIEZO1 agonist YODA1 to cultured reticulocytes (Fig. 5D), known to reduce the sensitivity threshold of the channel^51^, we observed reduced activity of the channel in cells with reduced cholesterol. These results provide validation of PIEZO1 interaction with cholesterol in human reticulocytes without the necessity of chemically depleting cholesterol, such as with MßCD, a method often used despite producing nonspecific effects and non-physiological cholesterol depletion^52^. In addition to its impact on mechanotransduction, the lipid composition of the membrane is also known to affect interactions with pathogens, with cholesterol playing a key role in the formation of membrane entry points for *Plasmodium falciparum*^53^ .We demonstrated how RET^Plasma^, with low cholesterol content, experience reduced invasion efficiency (Fig. 5E). Similarly to the other discussed assays, supplementation with cholesterol-rich lipids reversed the effect, rescuing *P. falciparum* invasion to levels observed in serum supplemented cultured reticulocytes, indicating a direct correlation with membrane cholesterol content.

Taken all together, these data underscore the importance of careful selection of lipid supplementation when conducting erythroid culture *ex vivo*. While both AB serum and plasma can both support erythropoiesis, they do so with distinct impacts on cell maturation, membrane composition, and downstream functionality. These findings have broad implications for the standardisation of *ex vivo* erythropoiesis protocols and the interpretation of functional assays, particularly when reticulocytes are used to study membrane-associated phenomena or host-pathogen interactions.

Future work will need to focus further on dissecting the donor-specific lipid metabolic responses and exploring additional omics-based characterisation to refine serum-free or defined culture systems to ensure that such cultures consistently yield physiologically relevant reticulocytes, especially if these are to be used in humans as a replacement for donor derived RBCs or used to deliver therapeutics.

## Materials and methods

### Source material

All human blood material was provided with written informed consent for research use given in accordance with the Declaration of Helsinki (NHSBT, Filton, Bristol). The research into the mechanisms of erythropoiesis was reviewed and approved by Bristol Research Ethics committee (REC Number 12/SW/0199).

### Antibodies, fluorescent dyes and small molecules

For flow cytometry several antibodies were used. BRIC71, targeting Band 3, is a mouse IgG1 antibody from IBGRL Research Products and was used at a 1:2 dilution. A purified mouse IgG1 isotype control (clone MG1-45) from Biolegend was used at a 1:50 dilution. To detect the transferrin receptor, an APC-conjugated anti-human CD71 antibody (clone CY1G4), a rat IgG2a, was used, at 1:50 dilution. For control, an APC-conjugated anti-mouse IgG1 antibody (clone RMG1-1) was employed, also at 1:50. CD49d was detected using a FITC-conjugated anti-human CD49d antibody (clone MZ18-24A9), a mouse IgG2b from Miltenyi Biotech. A FITC-conjugated mouse IgG2b isotype control (clone IS6-11E5.11) was also included, both at 1:50 dilution.

The following compounds were used for various cellular assays. Filipin III (Sigma-Aldrich, F4767) was applied at 50 µg/mL to stain cholesterol. Fluo-4 (Thermo Fisher Scientific, F14201), a calcium indicator, was used at a concentration of 5 µM. For DNA staining, Hoechst 33342 (Invitrogen, B2261) was used at 5 µg/mL. Thiazole Orange (Sigma-Aldrich, 390062) was employed at 0.1 µg/mL to label RNA. To activate PIEZO1, Yoda1 (Tocris, 5586) was used across a range of concentrations, from 0.156 to 10 µM.

### CD34^+^ culture

Peripheral blood mononuclear cells are isolated from apheresis cones^20^, followed by CD34^+^ magnetic cell isolation with CD34^+^ MicroBead kit (Miltenyi Biotec) according to manufacturer’s protocol. Cells were cultured as described by Kupzig et al.^26^. Isolated cells were seeded at 1 × 10^5^/mL in IMDM base medium (3% (v/v) Heat-inactivated (30 min at 56 °C) Human Male AB Serum (Sigma-Aldrich), 2 mg/mL Human Serum Albumin (HSA; Irvine-Scientific), 10 µg/mL insulin (Sigma-Aldrich), 3 U/mL heparin (Sigma-Aldrich), 0.2 mg/mL holotransferrin (Sigma-Aldrich), 3 U/mL erythropoietin (Epo; Bristol Royal Infirmary), and 100 µg/mL streptomycin (Sigma-Aldrich)). From days 0 to 8 medium was supplemented with 40ng/mL SCF and 1ng/mL IL-3, from days 8–13 only 40ng/mL SCF, and from day 13 onwards no supplementation. Where indicated AB serum and HSA were replaced with 5% (v/v) Octaplas (Octapharma) and/or supplemented with 40 mg/L Cholesterol rich lipid mix (L4646 Sigma). Cells were cultured until day 20. To obtain a pure population of reticulocytes, the cultures were filtered by leukofiltration. A leukocyte reduction filter (Macopharma) was pre-soaked and equilibrated with phosphate-buffered saline (PBS) and the cultured cell suspension was loaded into the filter followed by at least three volumes of PBS and allowed to pass through under gravity. The resulting flow-through was then centrifuged at 400 × g, for 15 min and the pelleted cells were resuspended in PBSAG (1 mg/ml Bovine Serum Albumin (BSA), 2 mg/ml glucose, in PBS) and kept at 4 °C if not used immediately.

### Native reticulocyte isolation (CD71+)

CD71^+^ cells were isolated from the red cell fraction of apheresis waste following density gradient centrifugation. The packed red cell layer was collected, washed 3 times in PBS and magnetic isolation performed using the CD71 MicroBeads cell isolation kit (Miltenyi Biotec) to enrich for CD71-expressing reticulocytes. Briefly, 500µL of packed red cells per donor were washed twice in MACS buffer (0.5% (w/v) BSA, 0.6% (v/v) Citrate-Phosphate-Dextrose solution (CPD), in PBS) and resuspended in 5mL buffer. 100µL of anti-CD71 beads were added, and the mixture incubated for 15 min at 4 °C. After centrifugation, cells were resuspended in 10mL cold MACS buffer and loaded a LS column (maximum 500µL packed red cells per column). At least 7 washes of 5mL MACS buffer were applied and then the cells were eluted in 5mL MACS buffer using the supplied plunger. Purity was assessed by flow cytometry by labelling cells with anti-CD71-APC antibody (Biolegend, San Diego, USA), and the isolated native reticulocytes stored in PBSAG at 4 °C if not used immediately. We highlight that because it is often difficult to isolate enough nRETs to perform all experiments on the same donors, nRETs were used in this manuscript to serve primarily as a contextual physiological reference rather than a universal comparator across all assays.

### Osmotic fragility assays

Filtered reticulocytes (1–2 × 10^5^/well) were incubated in decreasing NaCl concentrations (0.9–0%) for 10 min at 37 °C. Lysis was stopped by adding 4x volume of PBSAG. Live cells, considered as having a normal FSC/SSC profile as defined by the 0.9% NaCl control, were counted by flow cytometry using the MACSQuant10^23^. Representative gating strategies are shown in *Supplementary Fig. 2*. RBC osmotic fragility was quantified by fitting normalised haemolysis data to a variable-slope sigmoidal curve using GraphPad Prism, which can be seen in *Supplementary Fig. 3*. The half-maximal lysis concentrations (IC_50_) were derived from the best-fit values with 95% confidence intervals.

### Total cholesterol labelling

Cholesterol labelling^54^ was performed by initially fixing the cells in 1% paraformaldehyde and 0.0075% glutaraldehyde in PBSAG, at room temperature, for 15 min, followed by three washed in PBSAG. The fixed cells were incubated with 50 µg/mL filipin (from 25 mg/mL stock in DMSO, Filipin III from *S. filipinensis*, Sigma-Aldrich) in PBS for 45 min in the dark. Cells were washed twice in PBS and analysed on the Fortessa X20 flow cytometer using UV excitation.

### Automated rheoscopy

A total of 1 × 10^6^ cells were diluted in 200µL of a polyvinylpyrrolidone solution (viscosity, 28.1mPa·s; Mechatronics Instruments). Cell deformability distributions were assessed in an Automated Rheoscope and Cell Analyzer (ARCA) according to previously published protocols^17^. At least 2000 valid cells per sample were analysed.

### PIEZO1 responsivity assay

Serial dilutions of Yoda-1 (Tocris, Bristol, UK) were prepared from a 20mM stock, and the final assay concentrations used were 10µM, 5µM, 2.5µM, 1.25µM, 625nM, 312.5nM, 156.3nM, and vehicle only (DMSO). 2 × 10^5^ of filtered CD34-derived reticulocytes were resuspended in StemSpan containing 5µM of Fluo-4 AM (Thermo Fisher Scientific). Cells were incubated for 45 min at 37 °C, washed in PBSAG and resuspended in 100µL IMDM (Source BioScience UK Ltd or Sigma-Aldrich) supplemented with 2% FCS (Gibco). To streamline the assay, for each well 40µL were acquired for a basal fluorescence measurement followed by Yoda-1 addition, using a 100X concentrated stock solution (e.g. adding 0.6 µL of 1mM stock to obtain 10µM final concentration), gently mixing the sample, waiting 60 s, and measuring the post-PIEZO1 activation calcium influx. The calcium influx was measured using flow cytometry (MACSQuant Analyzer 10). Representative flow cytometry traces of intracellular Ca^2+^ responses are shown in *Supplementary Fig. 4.*

### Reverse transcription quantitative-PCR (RT-qPCR)

RNA was isolated from frozen pellets (CD34^+^ differentiation day 8) using a RNeasy kit (Qiagen), following supplier’s protocol. Total RNA concentration was measured using NanoDrop (ThermoFisher). Complementary DNA (cDNA) was generated using the QuantiTect Reverse Transcription Kit (Qiagen) following the indicated protocol. The RT-qPCR was done in 20µL reactions in a MicroAmpTM Optical 96-Well Reaction Plate (Applied Biosystems). Assuming a total conversion of RNA to cDNA, 10ng of cDNA was used as template with 10µL of PowerUpTM SYBRTM Green Master Mix (Applied Biosystems), 1µL of each primer (10µM) and 6µL nuclease-free water. Each sample was analysed in triplicate and a no template control was included. The RT-qPCR were performed using the QuantiStudio3™ RT PCR system (Applied Biosystems) under the Standard cycling mode (Primer Tm > 60 °C) as indicated by the manufacture’s protocol for SYBR green dyes. The relative gene expression was determined with the 2^− ΔΔCt^ method, with the normalisation to the geometric mean of two housekeeping genes, GAPDH and PABPC1. Primers (Eurofins) used: GAPDH fw GAGTCAACGGATTTGGTCGT; GAPDH rev TTGATTTTGGAGGGATCTCG; PABPC1 fw CCAGCTGCTCCTAGACCACCA; PABPC1 rev GCAGGACGTGGACCCATTGTC; HMGCR fw TGGGATGACTCGTGGCCCAGTT; HMGCR rev TGGCATCCCCTGACCTGGACTG; SQLE fw TAAGGAGCAGCTCGAGGCCAGG; SQLE rev CACCCGGCTGCAGGAATTCTCC; LDLR fw CCAACCTGAGGAACGTGGTCGC; LDLR rev AGTGCCCAGGACAGAGTCGGTC; SREBP1 fw TGTGGCGGCTGCATTGAGAGTG; SREBP1 rev GGGGTACTGAGCACGGACCAGT; SREBP2 fw TCGAGTCAGGTTCTGGGGGCTG; SREBP2 rev TGCCTCCAGAAGGTGACCGAGG.

### *P. falciparum* invasion assays

*P. falciparum* strain 3D7 parasites (BEI Resources) were maintained in human erythrocytes at 5% hematocrit using standard culture conditions^55^. As described in King et al.^56^, schizont stage parasites were purified using the Magnetic Cell Separation (MACS) system (Miltenyi Biotec) and added to wells of a round bottomed 96-well plate containing 1 × 10^6^ CD34-derived reticulocytes or RBCs. Parasitemias used ranged between 1 and 8%. Heparin (100 mU/µl final) was used as a negative control by inhibiting invasion. After ~ 18 h, invasion was quantified using flow cytometry by staining cells with SYBR Green (1:2000 in culture media; Sigma-Aldrich) for 30 min at 37 °C in the dark. Cells were centrifuged, SYBR Green containing media removed and the cell were fixed for 15 min at room temperature in 1% paraformaldehyde and 0.0075% glutaraldehyde in PBSAG, followed by three washed in PBSAG. Invasion was quantified based on SYBR green positivity using the heparin control to correct for background events within the invasion gate.

### Metabolomics

Metabolomics analyses were performed as previously described^21,57^. Cells were extracted at a concentration of 1 × 10⁶ cells/mL using ice-cold methanol: acetonitrile: water (5:3:2, v/v/v). Samples were vortexed for 30 min at 4 °C and then centrifuged at 12,000 g for 10 min at 4 °C to remove debris. The clarified supernatants were used for metabolite analysis, with 10 µL injected per run onto an ultra-high-pressure liquid chromatography system coupled to a high-resolution mass spectrometer (UHPLC-MS; Vanquish UHPLC and Q Exactive Orbitrap, Thermo). Separation of polar metabolites was achieved on a Phenomenex Kinetex C18 column (2.1 × 150 mm, 1.7 μm) at 45 °C, using a 5-minute gradient method under both positive and negative electrospray ionisation (conducted in separate runs) across a scan range of 65–975 m/z, following protocols previously established^57^.

Oxylipins were analysed using a Waters ACQUITY UPLC BEH C18 column (2.1 × 100 mm, 1.7 μm) at 60 °C. Mobile phase A consisted of 20:80:0.02 MeCN: water: formic acid, and mobile phase B of 20:80:0.02 MeCN: isopropanol: formic acid. Chromatographic separation in negative ion mode employed a 0.35 mL/min flow rate and the following gradient: 0% B (0–0.5 min), 25% B (1 min), 40% B (2.5 min), 55% B (2.6 min), 70% B (4.5 min), 100% B (4.6–6 min), and re-equilibration at 0% B (6.1–7 min). Mass spectrometric detection was performed in full scan mode (2 µscans) from 150 to 1500 m/z at 70,000 resolution, using a spray voltage of 4 kV, sheath gas at 45, and auxiliary gas at 15. Raw data (.raw) were converted to .mzXML format using RawConverter, then processed and analysed in Maven (Princeton University) using KEGG annotations and an internal library of reference standards. Quality control was monitored through the inclusion of technical replicates at the beginning, middle, and end of each analytical sequence, as previously described^58^.

### Lipidomics

Total lipids were extracted as previously described^21,59^: Cells were extracted in pre-chilled methanol at a concentration of 1 × 10^6^ cells/mL. After brief vortexing, samples were incubated at − 20 °C for 30 min to facilitate protein precipitation. Following incubation, extracts were centrifuged at 12,700 RPM for 10 min at 4 °C, and 80 µL of the resulting supernatant was transferred to fresh tubes for lipidomic analysis. Each extract (10 µL per injection) was analysed using a Thermo Vanquish UHPLC system coupled to a Q Exactive Orbitrap mass spectrometer, using a 5-minute lipid separation method. Chromatographic resolution was achieved on a Kinetex C18 column (30 × 2.1 mm, 1.7 μm; Phenomenex) maintained at 50 °C. Mobile phase A consisted of 25:75 MeCN: water with 5 mM ammonium acetate, while mobile phase B was composed of 90:10 isopropanol: MeCN, also with 5 mM ammonium acetate. The gradient was programmed as follows: 10% B at 0 min (0.3 mL/min), 95% B at 3.0 min (0.3 mL/min), held at 95% B until 4.2 min, dropped to 10% B at 4.3 min (0.45 mL/min), then equilibrated at 10% B from 4.9 to 5.0 min (flow rates: 0.4 then 0.3 mL/min). Data were acquired in both positive and negative electrospray ionisation modes in separate runs, scanning from 125 to 1500 m/z at 70,000 resolution. The source parameters included a spray voltage of 4 kV, sheath gas of 45, and auxiliary gas of 25. Data-dependent MS2 acquisition (ddMS^2^ was performed, selecting the top 10 most intense precursor ions for fragmentation. Raw files were processed using LipidSearch v5.0 (Thermo Scientific) for lipid identification and quantification.

### Proteomics

Proteomics analyses were performed as described^21,60^. Cells (10 µL) were lysed in 90 µL of distilled water. From each lysate, 5 µL was combined with 45 µL of 5% SDS, followed by vortexing. Samples were reduced with 10 mM dithiothreitol (DTT) at 55 °C for 30 min, cooled to room temperature, and then alkylated in the dark using 25 mM iodoacetamide for another 30 min. Following this, 1.2% phosphoric acid was added, and six volumes of binding buffer (90% methanol, 100 mM triethylammonium bicarbonate [TEAB], pH 7.1) were introduced to each sample. After gentle mixing, samples were loaded onto an S-Trap 96-well plate and centrifuged at 1500 × g for 2 min. The flow-through was collected and reapplied to the plate three times. Bound proteins were washed three times using 200 µL of binding buffer. For enzymatic digestion, 1 µg of sequencing-grade trypsin (Promega) in 125 µL of digestion buffer (50 mM TEAB) was added directly to the filters, and samples were incubated at 37 °C for 6 h. Peptides were sequentially eluted using 100 µL of each of the following solutions (with one repeat per buffer): 50 mM TEAB, 0.2% formic acid (FA), and 50% acetonitrile containing 0.2% FA. The eluted peptide fractions were combined, lyophilised, and resuspended in 500 µL of 0.1% FA. Desalting was performed using individual Evotips preloaded with each sample, which were washed with 200 µL of 0.1% FA and stored in 100 µL of 0.1% FA until analysis. Peptide separation was performed on an Evosep One system (Evosep, Odense, Denmark) using a PepSep analytical column (150 μm inner diameter, 15 cm) packed with ReproSil C18 resin (1.9 μm, 120 Å). The system was coupled to a timsTOF Pro mass spectrometer (Bruker Daltonics, Bremen, Germany) equipped with a Captive Spray nano-electrospray source. Acquisition was performed in PASEF mode, with a ramp time of 100 ms and 10 PASEF MS/MS scans per topN cycle. Full MS and MS/MS scans were acquired over an m/z range of 100–1700, with ion mobility scans between 0.7 and 1.50 Vs/cm². Precursor ions were isolated within a ± 1 Th window and fragmented using ion mobility-dependent collision energy ramping from 20 to 59 eV (positive mode). Low-abundance precursors (≥ 500 counts, < 20,000 counts) were scheduled for repeated acquisition unless dynamically excluded for 0.4 min.

### Database searching and protein identification

Raw MS/MS data were processed as mentioned previously^21^, by using MS Convert (ProteoWizard, version 3.0) to generate Mascot Generic Format (.mgf) files. Spectral matching was conducted with Mascot (v. 2.5) against the human UniProt database. The precursor and fragment ion mass tolerances were set to ± 15 ppm and ± 0.4 Da, respectively. Database searches assumed trypsin digestion, permitting up to one missed cleavage site. Variable modifications included protein N-terminal acetylation, lysine isopeptide bond formation (with associated ammonia loss), and pyroglutamate formation at the peptide N-terminus. Carbamidomethylation of cysteine residues was applied as a fixed modification.

Peptide and protein identifications were further validated using Scaffold (v. 4.8, Proteome Software, Portland, OR). Peptide identifications required a confidence level exceeding 95% based on the Peptide Prophet algorithm, while protein identifications were retained only if supported by at least two unique peptides and a minimum 99% probability score.

### Statistics

Data was organised and analysed using either Excel or GraphPad Prism 10 software. Unless stated otherwise, data is displayed as mean ± standard deviation (SD). Statistical analysis was completed where appropriate, first verifying normal distribution of the data using the Shapiro-Wilk normality test (significance level of 0.05). For non-parametric data sets, the Mann-Whitney U test was used to compare 2 groups and a Kruskal-Wallis test with Bonferroni correction was used when comparing 3 or more groups. For parametric data sets a Brown-Forsythe and Welch test was performed to test for differences between groups. *P* < 0.05 was set as statistical significance threshold (*p < 0.05, **p < 0.01, ***p < 0.001. ****p < 0.0001.

Statistical analyses of omics datasets were performed using MetaboAnalyst v5.0 and RStudio, following autoscaling (i.e., centring each variable by its mean and scaling by its standard deviation). Spearman correlation coefficients were calculated on the raw data, and visualised using line and volcano plots generated in RStudio. Multiple testing correction was applied using the false discovery rate (FDR) method to determine significance. Network and pathway enrichment analyses were carried out in OmicsNet v2.0, using as input the metabolites and proteins showing significant changes (FDR-adjusted *p* < 0.05).

### Limitations

This study has some methodological constraints that should be considered. Direct quantification of membrane cholesterol was not performed, and CRL supplementation was tested at only one concentration. Although lower CRL doses might mitigate the reduced deformability observed, optimisation of cholesterol repletion protocols has been addressed in other studies and was beyond the scope of this work. Filipin staining may also be affected by residual cholesterol micelles associated with cells after CRL treatment, potentially contributing to elevated MFI values, however the protocol used involved several washes before measurement to avoid such possibility.

The number of native circulating reticulocytes (nRETs) obtained from apheresis cones was limited, preventing their inclusion in all assays; yields of CD71 positive native reticulocytes that can be obtained using antibody conjugated magnetic bead isolation are low and expensive to achieve. For qRT-PCR, both GAPDH and PABPC1 were used as housekeeping genes. Although GAPDH can be sensitive to metabolic state, these two genes showed the most consistent expression across the differentiation stages analysed.

## Supplementary Information

Below is the link to the electronic supplementary material.

Supplementary Material 1

## Data Availability

The proteomics data set is available at MassIVE. The MassIVE identifier is MSV000094204, and can be accessed directly through the link [https://massive.ucsd.edu/ProteoSAFe/private-dataset.jsp?task=ab88082fec2b43b2952a16535b013127] . The relevant samples include cultured reticulocytes ( *cultured_reticulocytes_07* to *_15* ), with subsets as follows: 07-09 cultured in AB serum, 10-12 in plasma, and 13-15 in plasma supplemented with CRL. Samples *RBCs_16* to *_18* correspond to erythrocyte controls.The metabolomics and lipidomics data sets are available at the National Institutes of Health Common Fund’s National Metabolomics Data Repository website, the Metabolomics Workbench, [https://www.metabolomicsworkbench.org] for which it has been assigned study ID ST003108. Samples under Assay 01CF19 are pertinent to this manuscript, following the same subsets are detailed above (see last number on *local_sample_id* for correct sample identification).
