# Supplementary material for: Choice of lipid supplementation for in vitro erythroid cell culture impacts reticulocyte yield and characteristics
Source: Sci Rep. 2026 Jan 29;16:6632. doi: 10.1038/s41598-026-37229-z (PMC12914027; doi:10.1038/s41598-026-37229-z)
Supplement: Supplementary file 1 — Supplementary Material 1 [file 41598_2026_37229_MOESM1_ESM.pdf]

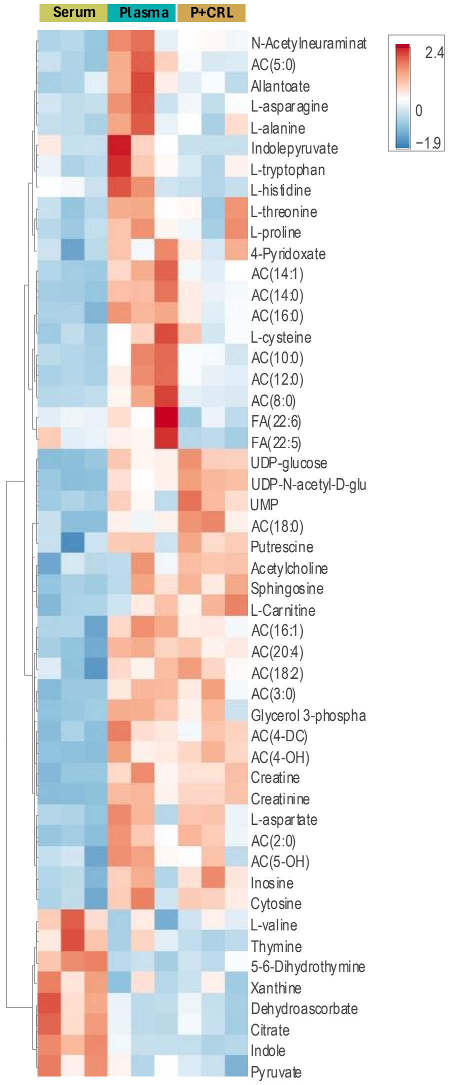

Supplemental Figure 1: Summary of metabolic differences in CD34-derived reticulocytes grown in the presence of Serum, Plasma, or Plasma supplemented with cholesterol-rich lipids

Serum, Plasma and Plasma+CRL compared with control RBCs (top 50 metabolites by ANOVA).

A

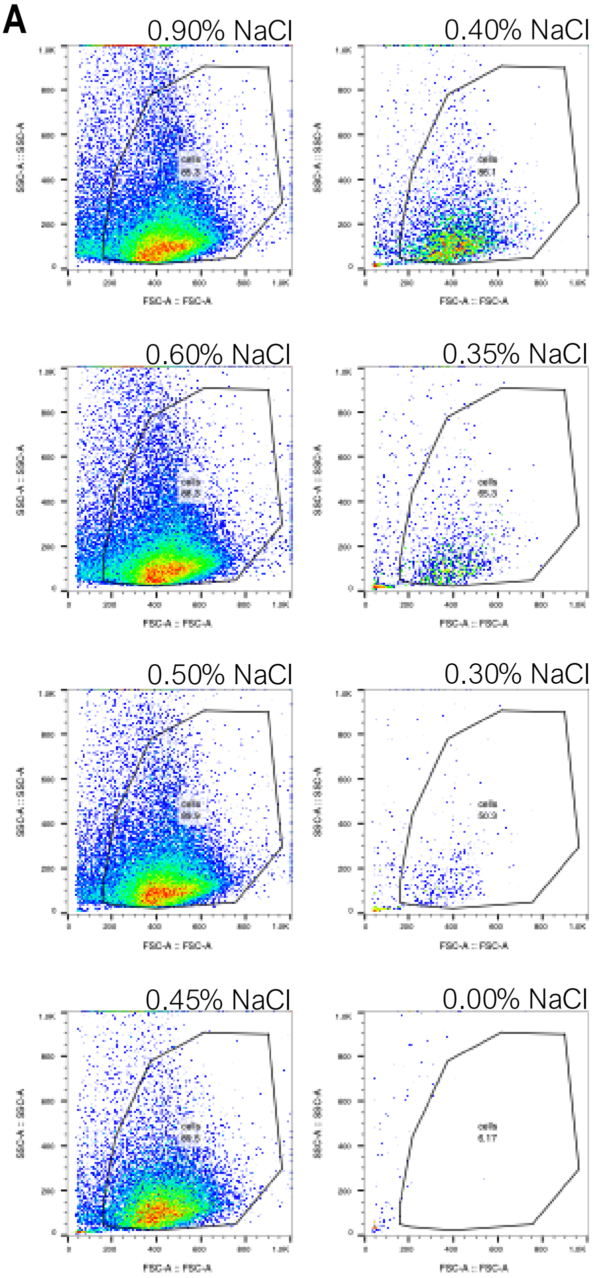

B

NaCl %	Cell count	Lysis (%)	Adjusted Lysis (%)
0.90%	80088	0.00%	0.00%
0.60%	82132	-2.55%	0.00%
0.50%	78529	1.95%	1.95%
0.45%	52125	34.92%	34.92%
0.40%	11593	85.52%	85.52%
0.35%	3402	95.75%	95.75%
0.30%	885	98.89%	98.89%
0.00%	14	99.98%	99.98%

C

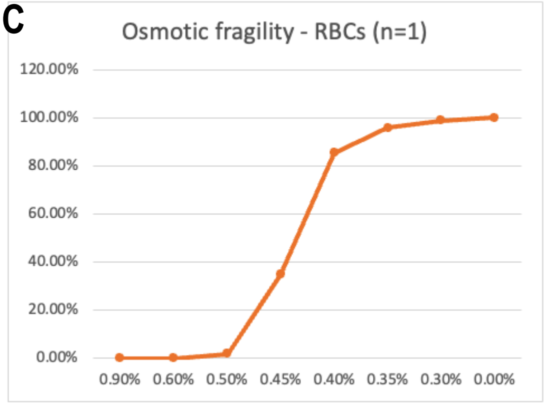

Supplemental Figure 2: Representative workflow and readout for the osmotic fragility assay.

A Flow cytometry dot plots of RBCs exposed to a titration of NaCl solutions ranging from 0.0% to 0.9%. A consistent gate around intact cells was applied to all samples. As NaCl concentrations decreases cell lysis increases. **B** Corresponding table summarizing absolute cell counts acquired at each NaCl as seen in (A). Percentage of lysis was calculated based on no lysis occurring at isotonic 0.9% NaCl. **C** Representative osmotic fragility curve generated from the data in (B), showing the percentage of lysed cells as a function of NaCl concentration.

A

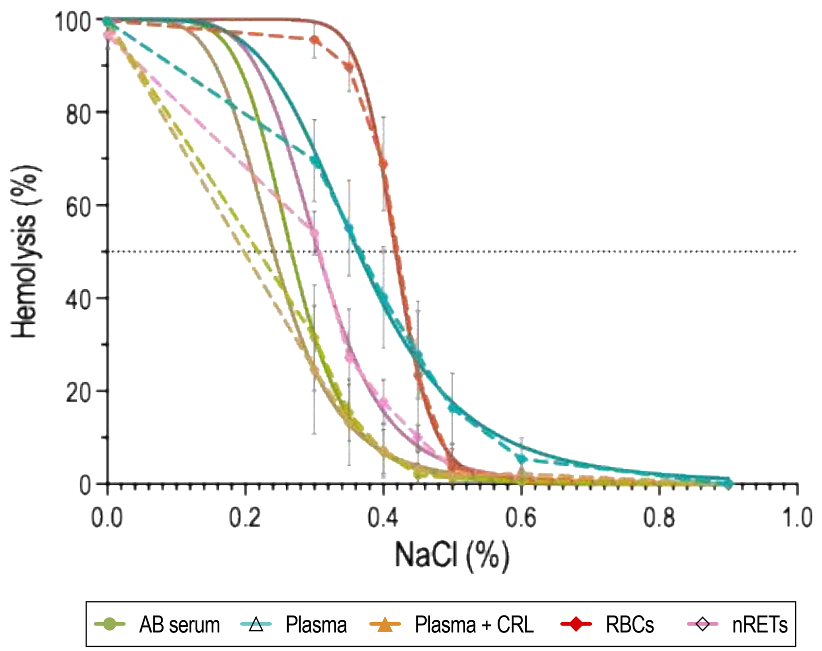

B

	Nonlin fit<br>Table of results	Plasma	Plasma + CRL	AB serum	RBCs	nRETs
1	**[Inhibitor] vs. normalized response -- Variable slope**					
2	**Best-fit values**					
3	IC50	0.3635	0.2411	0.2669	0.4178	0.3055
4	HillSlope	-4.834	-5.108	-6.499	-15.64	-6.248
5	logIC50	-0.4395	-0.6179	-0.5737	-0.3790	-0.5149
6	**95% CI (profile likelihood)**					
7	IC50	0.3559 to 0.3707	0.2175 to 0.2581	0.2570 to 0.2748	0.4143 to 0.4212	0.2981 to 0.3120
8	HillSlope	-5.373 to -4.338	-6.620 to -3.921	-7.636 to -5.538	-17.60 to -13.95	-7.238 to -5.392
9	logIC50	-0.4486 to -0.4309	-0.6624 to -0.5883	-0.5900 to -0.5610	-0.3827 to -0.3755	-0.5257 to -0.5058
10	**Goodness of Fit**					
11	Degrees of Freedom	94	94	94	134	44
12	R squared	0.9471	0.9613	0.9771	0.9700	0.9771
13	Sum of Squares	5450	3778	2280	7575	1097
14	Sy.x	7.614	6.339	4.925	7.519	4.992

Supplemental Figure 3. Osmotic fragility analysis and IC_{50} determination. **A** The dotted lines show mean heamolysis (%) determined across a NaCl dilution series (0.0-0.9%) for all conditions in the study (AB serum, Plasma, Plasma+CRL, RBCs, and native reticulocytes (nRETs)). Haemolysis was calculated from flow-cytometric intact-cell counts at each NaCl concentration relative to the isotonic control (0.9% NaCl) as exemplified in Supplemental Figure 2. The solid line shows the variable-slope sigmoidal fit used to calculate the half-maximal hemolysis concentration (IC_{50}). The adjacent table (**B**) summarizes the IC_{50} best-fit values, 95% confidence intervals (CI), and goodness-of-fit parameters derived from GraphPad Prism.

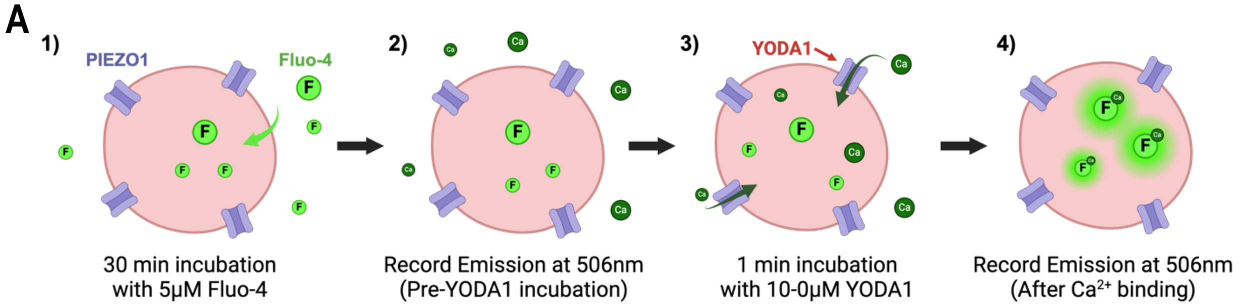

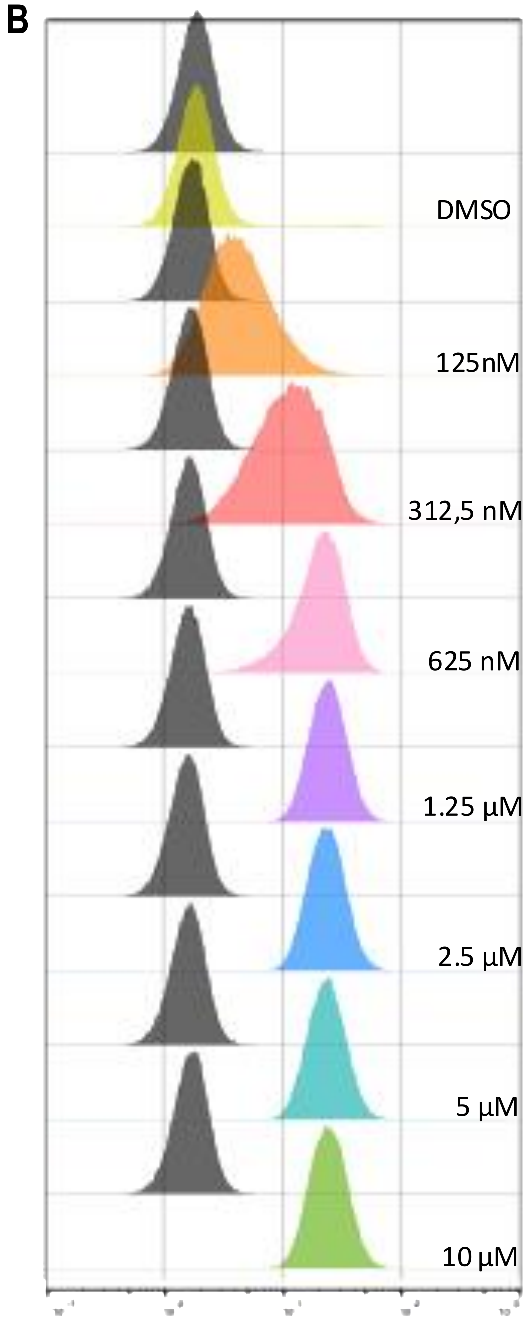

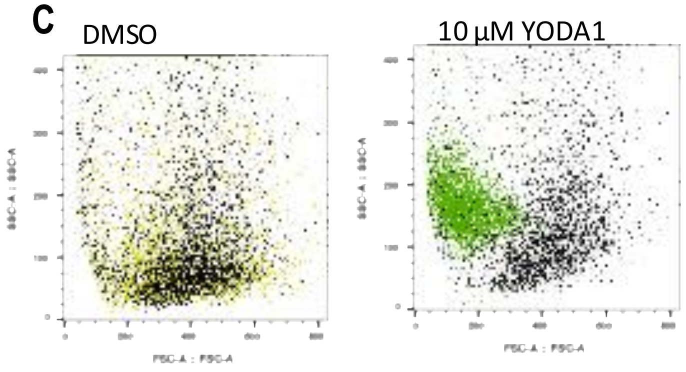

Supplemental Figure 4: Description of flow cytometry–based assay to determine PIEZO1 activity.

A Schematic overview of the assay. Cells were loaded with the Ca^{2+}-sensitive dye Fluo-4 AM, washed, and stimulated with the PIEZO1 agonist Yoda1 to induce Ca^{2+} influx. Increases in intracellular Ca^{2+} result in proportional increases in Fluo-4 fluorescence. (Created with BioRender) **B** Representative flow-cytometry histograms showing Fluo-4 fluorescence before stimulation (unstimulated baseline; black) and after treatment with increasing concentrations of Yoda1 (DMSO vehicle, 125 nM to 10 µM). Higher Yoda1 concentrations result in a clear right-shift of the fluorescence profile, consistent with increased PIEZO1-mediated Ca^{2+} entry.

C Corresponding FSC/SSC dot plots for the DMSO vehicle control and 10 µM Yoda1-treated cells. PIEZO1 activation induces characteristic changes in cell volume, visualised as a shift in forward scatter.
